# Vaccination Coverage and Associated Factors of Hepatitis B, Measles-Mumps-Rubella, Varicella, and Tetanus-Diphtheria-Acellular Pertussis Among Primary Health Care Workers in Qatar: A Retrospective Study (2020–2024)

**DOI:** 10.7759/cureus.86901

**Published:** 2025-06-28

**Authors:** Sameera Alhajri, Anees A Alyafei, Sandy Semaan, Maryam Al Muslemani, Asma Al Nuaimi

**Affiliations:** 1 Occupational Health and Safety, Primary Health Care Corporation, Doha, QAT; 2 Preventive Medicine, Primary Health Care Corporation, Doha, QAT; 3 Strategy and Planning, Primary Health Care Corporation, Doha, QAT; 4 Planning and Health Intelligence, Primary Health Care Corporation, Doha, QAT

**Keywords:** healthcare workers, hepatitis b vaccine (hbv), measles-mumps-rubella, primary health care, tetanus-diphtheria-pertussis (tdap), vaccination coverage

## Abstract

Background

Healthcare workers (HCWs) are at an increased risk of exposure to infectious diseases, making vaccination a cornerstone of occupational health and patient safety. Despite clear recommendations, vaccination coverage among HCWs remains suboptimal worldwide, with significant disparities influenced by sociodemographic and professional factors. There is a lack of published data on vaccine uptake among HCWs in Qatar, particularly within the Primary Health Care Corporation (PHCC). This study aimed to assess the vaccination coverage rates for hepatitis B virus (HBV), measles, mumps, and rubella (MMR), varicella, and tetanus, diphtheria, and acellular pertussis (Tdap) among HCWs at PHCC in Qatar from 2020 to 2024 and to identify factors associated with vaccination status.

Methods

A quantitative, retrospective analysis was conducted using data from the Electronic Medical Records System and Human Resources Database across 31 PHCC health centers. All HCWs employed for at least three months were included. Sociodemographic and career-related variables were extracted. Vaccination status for HBV, MMR, varicella, and Tdap was determined. Descriptive statistics summarized coverage rates, and chi-square tests assessed the associations between vaccination status and sociodemographic variables. A p-value was considered statistically significant at <0.005.

Results

Among 7,463 PHCC employees, the mean age was 40.82 ± 9.00 years, with 65.06% females and 72.18% non-Qatari nationals. Clinical HCWs comprised 64.24% of the workforce. Vaccination coverage was 16.75% for HBV, 5.86% for MMR, 1.76% for varicella, and 12.87% for Tdap. Coverage rates were significantly higher among younger age groups, females, non-Qatari nationals, and those with fewer years of service (p < 0.001 for most comparisons). Non-clinical HCWs had higher HBV coverage, while clinical HCWs had higher Varicella coverage. Educational qualification was associated with HBV and MMR coverage but not with varicella or Tdap.

Conclusions

Vaccination coverage among PHCC HCWs in Qatar is considerably lower than international targets, with significant disparities across age, gender, nationality, job role, years of service, and education. These findings underscore the need for targeted interventions to enhance vaccine uptake and address barriers among specific HCW subgroups, thereby improving occupational and patient safety.

## Introduction

Vaccination plays a vital role in occupational health and patient safety, particularly for healthcare workers (HCWs) who are at a heightened risk of exposure to infectious diseases due to their frontline positions and regular interactions with patients [1]. Ensuring high vaccination coverage among HCWs not only protects their own health but also reduces the risk of disease transmission to vulnerable patient populations and helps maintain continuity of healthcare services during outbreaks [2].

There is a wide range of vaccinations recommended for HCWs, including the hepatitis B vaccine (HBV), measles, mumps, and rubella vaccine (MMR), varicella, and tetanus, diphtheria, and acellular pertussis vaccine (Tdap). Vaccination coverage for key vaccines varies significantly worldwide due to differences in healthcare infrastructure, policy priorities, and socioeconomic factors [3].

For the HBV, uptake among HCWs shows significant global disparities. In the United States, around 75% of HCWs have received the full three-dose HBV series. In Australia and New Zealand, the rate is even higher at (77%). However, vaccination coverage is significantly lower in several African countries, with about 12.8% of healthcare workers in Nigeria having received at least one dose and only 10.5% being fully vaccinated [4,5].

For the MMR, immunity among HCWs also varies. A study from the UK found serological positivity rates among new HCWs to be 88.2% for measles, 68.8% for mumps, and 93.9% for rubella [6], while the percentage was less in Turkey, reaching 28% [7].

Regarding the varicella vaccine, while specific uptake data among HCWs are less frequently reported, in Italy, it was reported to be 20% to 30%, while globally, it was reported to be as low as 5% [8,9]. For Tdap coverage among HCWs, it is generally low. In a large university hospital in Southern Italy, only 34.5% of HCWs had received a Tdap booster in the past 10 years. A systematic review found an average coverage of just 40% across studies, with the highest reported rate being 63.9% [10].

Despite clear recommendations and the demonstrated benefits of vaccination, including reduced absenteeism, lower nosocomial infection rates, and decreased mortality, vaccine uptake among HCWs often falls short of targets [11]. The low vaccination coverage among HCWs is driven by a combination of knowledge gaps, organizational shortcomings, vaccine hesitancy, convenience barriers, complacency, and sociodemographic factors, including age, gender, nationality, years of work, job roles, educational level, and others [12,13].

The Primary Health Care Corporation (PHCC) serves as the foremost governmental provider of primary care services in Qatar, operating a network of 31 health centers strategically located throughout the country. These facilities deliver a comprehensive array of services, supported by a workforce of 7,463 professionals. The staff encompasses both clinical and non-clinical HCWs to ensure the delivery of safe and high-quality medical services. The staff routinely offered recommended vaccinations, including MMR, varicella, and Tdap. Additionally, HBV is highly encouraged as part of the first licensing process, with provisions for those who are unvaccinated or show a low antibody titer. There are currently no mandated policies governing the administration of MMR, varicella, HBV, and Tdap vaccinations for HCWs at PHCC. Instead, PHCC proactively engages in educational efforts and provides accessible vaccination services to all HWCs at no cost.

Given the absence of published data regarding vaccination coverage among HCWs within the PHCC, the objective of the current study was to evaluate the current vaccination coverage of HCWs employed at PHCC in Qatar for the vaccines, including HBV, MMR, varicella, and Tdap, beginning from the year 2020. Additionally, the study aims to identify the factors associated with the vaccination status of HCWs employed at PHCC in Qatar since 2020.

## Materials and methods

Study design and setting

This quantitative, retrospective study analyzed HCWs' vaccination status at the PHCC in Qatar. Data were obtained from the Electronic Medical Records System (EMRS) across 31 health centers and the Human Resources Database (HRD). The study analyzed available data from January 2020 to July 2024.

Study population

The study population included all HCWs employed by PHCC who had been in service for at least three months. Both female and male staff were included. Eligible participants comprised clinical professionals, allied health professionals, administrative staff engaged at headquarters or community health centers, and workers involved in direct patient care or exposed to blood and body fluids. Outsourced staff, temporary workers, and employees with less than three months of service were excluded.

Sampling strategy

A consecutive sampling method was used, which included all eligible HCWs during the specified study period. This approach ensured complete inclusion without predefined selection criteria, thereby minimizing sampling bias.

Data collection

Data were anonymously collected from two institutional sources: the HRD and the EMRS. Sociodemographic variables included age (completed years), gender (female or male), and nationality (Qatari or non-Qatari). Career-related variables included job role (clinical or non-clinical), years of experience (completed years), and educational qualifications (below bachelor's degree versus bachelor's degree or higher).

Vaccination records for HBV, MMR, varicella, and Tdap vaccines were extracted from the EMRS, including vaccination status (vaccinated or not) and the number of doses administered. All data were securely stored in a password-protected Microsoft Excel® file (Microsoft Corporation, Redmond, WA), with access restricted to authorized research team members only.

Quality measures

The study received ethical approval from the PHCC Institutional Review Board (IRB), ensuring adherence to established research ethics guidelines. All HCW data were anonymized to protect confidentiality.

A standardized data extraction protocol, approved by the IRB, was developed to guide the systematic identification and recording of information from the HRD and EMRS databases. The research team, led by the principal investigator, underwent training to ensure accuracy and consistency in data collection.

Multiple team members conducted regular reliability checks, with discrepancies resolved by consensus or escalated to the principal investigator. Routine audits and validation procedures were performed by cross-referencing extracted data with original EMRS records to confirm accuracy and adherence to the study protocol.

Data analysis

Descriptive statistics were used to calculate the characteristics of the study participants and their vaccination coverage rates in percentages. Continuous variables were presented as means and standard deviations, while categorical variables were presented as frequencies and percentages.

A chi-square test was also used to assess the relationship between vaccination status and sociodemographic factors. All statistical assessments were conducted using SPSS Statistics for Windows, Version 29.0.2.0 (IBM Corp., Armonk, NY), with a p-value of <0.05 considered statistically significant.

## Results

Due to the large dataset we collected, we decided to divide the analysis into two separate research studies to ensure each topic received appropriate depth and focus. The same dataset was used to analyze vaccination coverage for COVID-19 and seasonal influenza, which was recently published, representing age (Table 1) and sociodemographic characteristics (Table 2). The total dataset comprised 7,463 employees from PHCC, which was analyzed to assess the age of HCWs. The average age of these employees was 40.82 ± 9.00 years. The most prevalent age group was identified as individuals aged 19 to 39 years, who represented 48.69% of the total HCWs, while approximately one-third (33.93%) of the HCWs fell in the age range of 40 to 49 years. Further analysis of the PHCC workforce revealed that 4,856 (65.06%) employees were female. The nationality analysis showed that most employees were non-Qatari, 5,387 (72.18%).

**Table 1:** The age distribution of the Primary Health Care Corporation workforce 2024

Age group	n (%)	Mean ± SD
19-39	3,634 (48.69)	30.79 ± 5.10
40-49	2,532 (33.93)	44.67 ± 2.37
50-59	1,109 (14.86)	53.90 ± 2.98
≥60	188 (2.52)	62.08 ± 2.10
Total	7,463 (100)	40.82±9.00

**Table 2:** Characteristics of the PHCC workforce 2024 (N= 7,463). HCW, healthcare workers; PHCC, Primary Health Care Corporation

Sociodemographics	n (%)
Gender	
Females	4,856 (65.06)
Males	2,607 (34.93)
Nationality	
Qatari	2,076 (27.82)
Non-Qatari	5,387 (72.18)
Job role	
Clinical HCW	4,794 (64.24)
Non-clinical HCW	2,669 (35.75)
Years in PHCC	
0 to 10	5,283 (70.79)
11 to 20	1,541 (20.65)
More 21	639 (8.56)
Qualification	
Bachelor’s degree and above	5,562 (74.53)
Less than a bachelor’s degree	1,901 (25.47)
Total	7,463 (100.00)

The workforce was divided into two main job categories: clinical HCW, who accounted for 64.24% of the total PHCC workforce and were directly engaged in clinical or healthcare-related roles, and non-clinical HCW, who comprised 2,669 (35.75%) of the total PHCC workforce, where most of them (70.79%) had worked for 10 years or less. Regarding educational qualifications, out of 7,463 employees, 5,562 (74.53%) individuals held a bachelor’s degree or higher (Table 2).

**Figure 1:**
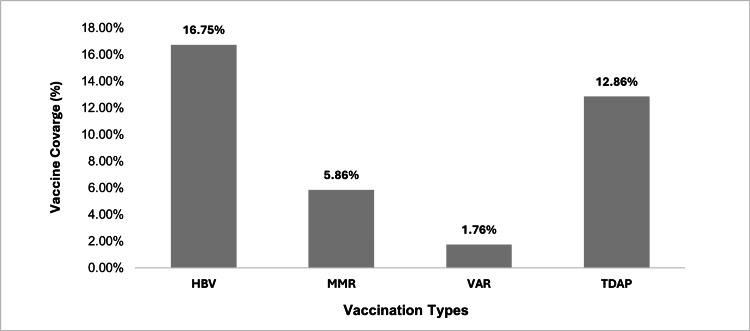
HBV, MMR, VAR, and Tdap vaccines coverage rate among healthcare workers of Primary Health Care Corporation, Qatar, 2024. HBV, hepatitis B vaccine; MMR, measles, mumps, and rubella; Tdap, tetanus, diphtheria, and acellular pertussis; VAR, varicella

Further analysis revealed the vaccination coverage among 7,463 staff members of the PHCC for HBV 1250 (16.75%), MMR 437 (5.86%), varicella 131 (1.76%), and Tdap 960 (12.87%), as illustrated in Figure 1.

Table 3 presents the relationship between various sociodemographic variables and vaccination coverage for HBV, MMR, varicella, and Tdap among HCWs at the PHCC in Qatar in 2024. The associations are evaluated using the chi-square (χ²) test, with corresponding p-values indicating statistical significance at <0.05.

**Table 3:** Association between sociodemographic and vaccination coverage for HBV and MMR among PHCC HCWs, Qatar, 2024 (N=7,463). Chi-square test p-value < 0.005 HBV, hepatitis B vaccine; HCW, healthcare worker; MMR, measles, mumps, and rubella; PHCC, Primary Health Care Corporation; Tdap, tetanus, diphtheria, and acellular pertussis

Vaccine	Variable	Category	No. vaccinated (%)	χ²-test	P-value
HBV	Age (completed years)	19-39	521 (6.98)	24.58	<0.001
		40-49	230 (15.93)		
		50-59	81 (13.13)		
		≥60	6 (7.50)		
	Gender	Females	838 (15.80)	2.47	0.1089
		Males	412 (17.26)		
	Nationality	Qatari	198 (9.54)	106.56	<0.001
		Non-Qatari	1,052 (19.52)		
	Job role	Clinical HCW	651 (13.06)	146.11	<0.001
		Non-clinical HCW	599 (24.18)		
	Years in PHCC	0-10	961 (18.19)	31.33	<0.001
		11-20	207 (13.43)		
		≥21	82 (12.83)		
	Qualification	Bachelor’s degree and above	1,106 (19.88)	93.39	<0.001
		Less than a bachelor’s degree	314 (16.45)		
MMR	Age (completed years)	19-39	304 (4.07)	165.59	<0.001
		40-49	81 (5.61)		
		50-59	9 (1.46)		
		≥60	0 (0.00)		
	Gender	Females	394 (8.11)	127.41	<0.001
		Males	43 (1.65)		
	Nationality	Qatari	152 (2.04)	10.85	<0.001
		Non-Qatari	285 (3.82)		
	Job role	Clinical HCW	215 (2.90)	108.98	<0.001
		Non-clinical HCW	222 (2.97)		
	Years in PHCC	0-10	358 (4.80)	46.56	<0.001
		11-20	65 (0.87)		
		≥21	14 (0.19)		
	Qualification	Bachelor’s degree and above	331 (5.95)	34.58	<0.001
		Less than a bachelor’s degree	157 (8.26)		

The analysis of HBV vaccination coverage revealed that the highest rate was in the age group of 19-39 years, with 676 (6.98%) vaccinated individuals, while the lowest was in those aged 60 and above, with 22 (7.50%) vaccinated. This significant disparity (p < 0.001) suggests that younger HCWs are more likely to receive the vaccine. Gender differences showed no significant variation in rates, with females at 15.80% and males at 17.26% (p = 0.1089). However, non-Qatari HCWs had a higher vaccination rate (19.52%) compared to Qatari HCWs (9.54%) (p < 0.001).

Non-clinical HCWs also showed higher vaccination rates (24.18%) compared to clinical HCWs (13.06%), which was significant (p < 0.001). Furthermore, HCWs with 0-10 years of service had higher rates (18.19%) than those with more experience, with a significant difference (p < 0.001). Lastly, HCWs with bachelor’s degrees or higher had relatively better coverage (19.88%) compared to those with lower qualifications (16.45%), also significant (p < 0.001).

The analysis of MMR vaccination coverage reveals that the highest rates were found in the age group of 19-39 years (4.07%). A significant decline was noted in older age groups, with no coverage in those aged 60 and above (p < 0.001). Additionally, a considerable gender disparity was observed, with females showing a higher vaccination rate of 8.11% compared to 1.65% for males (p < 0.001).

In terms of nationality, non-Qatari HCWs exhibited higher coverage at 3.82% compared to their Qatari counterparts, who had a coverage rate of 2.04% (p < 0.001). Furthermore, while vaccination coverage among clinical and non-clinical HCWs was similar, this association was statistically significant (p < 0.001). Regarding years of service, newer employees in the PHCC with 0-10 years of experience had a much higher vaccination rate of 4.80% compared to those with longer tenure (p < 0.001). Lastly, individuals possessing qualifications less than a bachelor's degree demonstrated higher coverage (8.26%) than those with a bachelor's degree or higher, who had a rate of 5.95% (p < 0.001).

Similarly, varicella vaccination coverage exhibited a vaccination rate within the age group of 19-39 years of 1.18%, while older HCWs showed negligible rates; this difference is highly significant (p < 0.001). Gender differences were also apparent, as females have a coverage rate of 2.18%, significantly higher than the 0.96% observed in males (p < 0.001). Additionally, the analysis indicated no statistically significant relation in coverage between Qatari and non-Qatari HCWs, with a p-value of 0.931 (Table 4).

**Table 4:** Association between sociodemographic and vaccination coverage for varicella, tetanus, diphtheria, and acellular pertussis, among PHCC HCWs, Qatar, 2024 (N=7,463). Chi-square test p-value < 0.005 HCW, healthcare worker; PHCC, Primary Health Care Corporation; Tdap, tetanus, diphtheria, and acellular pertussis

Vaccine	Variable	Category	No. vaccinated (%)	(χ²-test)	P-value
Varicella	Age (completed years)	19-39	88 (1.18)	96.88	<0.001
		40-49	13 (0.90)		
		50-59	5 (0.81)		
		≥60	0 (0.00)		
	Gender	Females	106 (2.18)	14.03	<0.001
		Males	25 (0.96)		
	Nationality	Qatari	36 (0.50)	0.00	0.931
		Non-Qatari	95 (1.27)		
	Job role	Clinical HCW	81 (1.09)	6.33	0.009
		Non-clinical HCW	50 (0.67)		
	Years in PHCC	0-10	115 (1.54)	32.51	<0.001
		11-20	14 (0.19)		
		≥21	2 (0.03)		
	Qualification	Bachelor’s degree and above	103 (1.85)	8.58	0.284
		Less than a bachelor’s degree	44 (2.31)		
Tdap	Age (completed years)	19-39	684 (9.17)	456.53	<0.001
		40-49	127 (8.80)		
		50-59	21 (3.40)		
		≥60	5 (6.25)		
	Gender	Females	837 (17.24)	236.04	<0.001
		Males	123 (4.72)		
	Nationality	Qatari	98 (1.31)	169.13	<0.001
		Non-Qatari	862 (11.55)		
	Job role	Clinical HCW	677 (9.07)	0.31	0.5551
		Non-clinical HCW	283 (3.79)		
	Years in PHCC	0-10	831 (11.13)	143.41	<0.001
		11-20	109 (1.46)		
		≥21	20 (0.27)		
	Qualification	Bachelor’s degree and above	934 (16.79)	218.48	<0.001
		Less than a bachelor’s degree	170 (8.94)		

Further analysis revealed that clinical HCWs show higher vaccination coverage at 1.09% compared to 0.67% for non-clinical workers, a difference that is statistically significant (p = 0.009). Investigating years of service within the PHCC, the subgroup with 0-10 years of service has the highest coverage at 1.54%, also yielding highly significant results (p < 0.001). In contrast, no significant differences in vaccination coverage were found when evaluated by qualification, as indicated by a p-value of 0.284.

The Tdap vaccination coverage varied significantly across different demographics. The highest coverage was observed among the age groups of 19-39 (9.17%) and 40-49 (8.80%) years, with significantly lower rates in older age groups (p < 0.001). Gender played a crucial role as well, with females exhibiting higher coverage at 17.24% compared to males at (4.72%) (p < 0.001). Additionally, nationality had an impact on vaccination rates, as non-Qatari (HCWs) reported significantly higher coverage (11.55%) compared to their Qatari counterparts (1.31%) (p < 0.001).

Regarding job role, no statistically significant difference was observed between clinical and non-clinical HCWs (p = 0.5551). Interestingly, newer staff members, with 0-10 years of experience in the PHCC, had a higher vaccination coverage of 11.13% (p < 0.001). Moreover, qualifications were also a determining factor, as individuals holding a bachelor’s degree or higher displayed higher coverage rates at 16.79% compared to 8.94% for those with lower qualifications (p < 0.001).

Most associations were statistically significant (p < 0.001), except for gender, with HBV and MMR, nationality, job role, qualification in varicella, and job role in Tdap.

In the multivariate analysis, several predictors were significantly linked to vaccine uptake among HCWs (Table 5). Those aged 50 years and older showed lower vaccination rates for MMR and Tdap, while female HCWs consistently had higher vaccination rates across all four vaccines. Nationality played a variable role in the outcomes; non-Qatari staff were less likely to receive the HBV and Tdap vaccines, but they were more inclined to get vaccinated for MMR and varicella. Interestingly, non-clinical HCWs had notably lower odds of being vaccinated for HBV, although the differences for the other vaccines were not statistically significant. Additionally, a shorter duration of service (less than 10 years) was associated with reduced uptake of HBV, Tdap, and varicella vaccines. On the other hand, education level did not show a significant connection to vaccine uptake in any of the models examined. These findings highlight that demographic and occupational characteristics significantly influence vaccination coverage, which should be taken into account when designing targeted public health interventions.

**Table 5:** Multivariate logistic regression analysis of predictors of vaccine uptake among HCWs at the Primary Health Care Corporation. P-value < 0.005 aOD, adjusted odds ratio; CI, confidence interval; HBV, hepatitis B vaccine; HCW, healthcare workers; MMR, measles, mumps, and rubella; Tdap, tetanus, diphtheria, and acellular pertussis

Predictor variable	HBV aOD [95% CI]	P-value	MMR aOD [95% CI]	P-value	TDAP aOD [95% CI]	P-value	VAR aOD [95% CI]	P-value
Age ≥50 vs <50	0.78 [0.61, 1.01]	0.067	0.22 [0.11, 0.45]	<0.001	0.45 [0.28, 0.72]	0.001	0.29 [0.07, 1.25]	0.097
Female vs male	0.74 [0.61, 0.90]	0.002	0.21 [0.13, 0.33]	<0.001	0.25 [0.18, 0.35]	<0.001	0.71 [0.35, 1.42]	<0.001
Non-Qatari vs Qatari	0.39 [0.32, 0.47]	<0.001	1.64 [1.22, 2.21]	0.001	0.32 [0.25, 0.42]	<0.001	1.99 [1.06, 3.74]	0.033
Non-clinical vs Clinical HCW	0.57 [0.45, 0.71]	<0.001	0.75 [0.47, 1.20]	0.228	0.75 [0.54, 1.03]	0.077	0.55 [0.21, 1.47]	0.230
Years of service <10 vs ≥10	0.76 [0.63, 0.93]	0.008	0.77 [0.55, 1.08]	0.128	0.51 [0.38, 0.70]	<0.001	0.39 [0.17, 0.90]	0.027
Bachelor's or above vs below	0.91 [0.69, 1.21]	0.524	0.84 [0.49, 1.46]	0.540	1.12 [0.73, 1.73]	0.610	0.63 [0.20, 1.98]	0.428

## Discussion

The present study on vaccination coverage among PHCC HCWs in Qatar revealed notably low uptake rates for HBV (16.75%), MMR (5.86%), varicella (1.76%), and Tdap (12.87%). These rates are considerably below international targets and are accompanied by significant disparities across age, gender, nationality, job role, years of service, and educational level.

Globally, HCW vaccination coverage is higher for most vaccines, such as HBV coverage in the USA and Australia, which exceeds 75%, while in some African countries, it remains below (20%) [14,15]. The MMR seropositivity among HCWs in Japan is above 90%, and even in Turkey, it is higher than the rates observed in this study [16,17]. Varicella and Tdap coverage rates are also higher in European and North American studies, with Tdap booster rates reaching up to 63.9% in some settings [18].

The low coverage in Qatar mirrors findings from other regions where institutional, behavioral, and logistical barriers persist. For instance, a meta-analysis of influenza vaccination among HCWs found an overall global rate of 41.7%, with significant regional disparities and similar influencing factors, including age, education, department, occupation, and risk awareness [19]. Studies from Africa and Asia also report that younger age, female gender, non-national status, fewer years of service, and higher education are associated with higher vaccine uptake, echoing the findings from Qatar [20,21].

In terms of age, younger HCWs in Qatar showed higher vaccination rates, consistent with global findings that younger staff are more likely to be vaccinated, possibly due to recent onboarding protocols or greater exposure to institutional mandates and education. However, some studies on HPV and COVID-19 vaccines have found higher uptake among older HCWs, likely due to increased risk perception and comorbidities [22,23]. Another possible explanation could be that younger workers were more knowledgeable and resilient in accepting the vaccination compared to older HCWs, who typically demonstrate more resistance to vaccines.

Female HCWs generally had higher coverage rates for MMR, varicella, and Tdap in Qatar, a trend also observed in other studies, particularly for COVID-19 and HPV vaccines [24]. Some research, however, reports no overall gender difference in acceptance but highlights gender-specific motivations for hesitancy or acceptance, such as concerns about fertility or reproductive health among women [25].

Non-Qatari HCWs had higher vaccination rates for HBV, MMR, and Tdap. This may reflect stricter licensing requirements for expatriates or greater compliance with institutional policies. Similar patterns are seen in other Gulf and Asian countries with large expatriate healthcare workforces [26].

From the job role and years of service perspectives, non-clinical HCWs in Qatar had higher HBV coverage, while clinical staff had higher varicella coverage. Newer employees (0-10 years of service) consistently showed higher vaccination rates across all vaccines, suggesting that onboarding and recent policy changes are effective at improving initial compliance but may not sustain long-term immunity. This finding is supported by studies from Italy and Uganda, where mandatory or actively promoted vaccination policies during onboarding increased uptake [27,28].

Higher educational attainment was associated with better HBV and Tdap coverage but not with varicella. This aligns with evidence that education increases risk perception and vaccine literacy, though the effect varies by vaccine and setting. The significance of these factors is supported by evidence from recent literature: access to information, including correct information and targeted communication, has been repeatedly shown to increase vaccine uptake. Studies from Vietnam and the USA have found that interventions such as active recall, educational sessions, and reminders significantly improve coverage [29,30].

The low vaccine coverage observed in this study can also be attributed to institutional and policy barriers, as well as social and behavioral obstacles. Critical institutional and policy barriers include the presence of mandatory vaccination policies, ease of access to vaccines, and the level of institutional support [12]. Meanwhile, social and behavioral issues, such as vaccine hesitancy, misconceptions about vaccines, and a sense of complacency, pose significant challenges, particularly among older staff members and those who have been with the organization for a longer period [31]. These individuals may not be affected by new onboarding protocols and often perceive their risk of illness to be lower, contributing to their reluctance to get vaccinated [32].

This study presents one of the first comprehensive assessments of vaccination coverage among HCWs in Qatar's PHCC, covering a large and representative workforce of over 7,000 individuals across 31 health centers. The use of routinely collected data from two robust institutional sources - the EMRS and HRD - enhances data completeness and minimizes recall bias. By stratifying results according to key sociodemographic and occupational variables, the study provides detailed insights into disparities in vaccine uptake, enabling targeted public health interventions. Additionally, the study applies rigorous statistical analysis to explore significant associations, reinforcing the validity of its conclusions.

Despite its strengths, the study's retrospective design inherently limits its ability to establish causal relationships between sociodemographic and professional factors and vaccination uptake. The reliance on EMR and HRD may obscure vaccinations administered outside of PHCC or those inadequately documented, potentially underestimating actual coverage rates. Furthermore, the study lacks qualitative data regarding vaccine hesitancy, beliefs, and institutional barriers, which could enrich the understanding of the low vaccination uptake.

The classification of vaccination status as binary (vaccinated or not) fails to distinguish between partial and complete vaccine series, which is particularly relevant for multi-dose regimens such as HBV vaccination. This limitation may result in misestimations of true immunization coverage. Additionally, the absence of serological testing to confirm immunity status means that the presence of protective antibodies among HCWs with undocumented vaccination histories could not be verified.

The study does not account for personal health conditions, contraindications, or prior natural infections that may have influenced vaccine eligibility and engagement. Lastly, while the dataset is comprehensive, the absence of qualitative insights regarding HCW attitudes, perceptions, and barriers to vaccination restricts a thorough understanding of the behavioral and psychosocial factors affecting vaccine uptake, thereby limiting the insights that can be derived from the findings.

## Conclusions

Vaccination coverage among PHCC HCWs in Qatar is substantially lower than international benchmarks, with notable disparities by age, gender, nationality, job role, years of service, and educational background. The findings are consistent with international evidence, which highlights the importance of onboarding protocols, targeted education, and institutional mandates in improving vaccine uptake. Younger, female, non-Qatari, and newly employed HCWs are more likely to be vaccinated, likely due to greater exposure to recent policy interventions and educational campaigns.

Persistent gaps in coverage, especially among older, male Qatari staff, and those with longer service, underscore the need for comprehensive, ongoing strategies. These should include regular catch-up programs, strengthened onboarding protocols to verify and administer all required vaccines, EMR-triggered reminders, regular staff education, and policy alignment with global standards. Addressing both institutional and behavioral barriers is crucial for enhancing vaccine uptake and safeguarding both HCWs and patients.

## References

[1] Bianchi FP, Stefanizzi P, De Maria L (2022). Vaccination offer during the Occupational Health Surveillance Program for Healthcare Workers and suitability to work: an Italian retrospective cohort study. Vaccines (Basel).

[2] Clari M, Albanesi B, Comoretto RI (2024). Effectiveness of interventions to increase healthcare workers' adherence to vaccination against vaccine-preventable diseases: a systematic review and meta-analysis, 1993 to 2022. Euro Surveill.

[3] Kolobova I, Nyaku MK, Karakusevic A, Bridge D, Fotheringham I, O'Brien M (2022). Vaccine uptake and barriers to vaccination among at-risk adult populations in the US. Hum Vaccin Immunother.

[4] Mohanty SS, Panda PS, Samantara C, Samantaray A (2022). Coverage of hepatitis-B vaccination among the healthcare providers of a tertiary care hospital in Odisha: a cross-sectional study. Curr Med Issues.

[5] Atekoja O, Adeniyi O, Ladipo M, Omitogun O, Adeniyi O, Richard A, Ogundare T (2025). Determinants and uptake of hepatitis B virus vaccination among healthcare workers: a survey in a government-owned teaching hospital Nigeria. Nurs Health Sci J.

[6] Basu S, Giri P, Adisesh A, McNA R (2014). Healthcare workers and measles-mumps-rubella (MMR) status: how worried should we be about further outbreaks?. Epidemiol Infect.

[7] Kumbul H, Önal Ö (2023). Evaluation of immunization status of healthcare workers and factors affecting immunization in Suleyman Demirel University Research and Training Hospital. Med J SDU.

[8] Genovese C, Picerno IA, Trimarchi G (2019). Vaccination coverage in healthcare workers: a multicenter cross-sectional study in Italy. J Prev Med Hyg.

[9] Riccò M, Ferraro P, Zaffina S (2024). Immunity to varicella zoster virus in healthcare workers: a systematic review and meta-analysis (2024). Vaccines (Basel).

[10] Mercogliano M, Fiorilla C, Esposito F (2023). Knowledge and attitude factors associated with the prevalence of Tdap (tetanus, diphtheria, and acellular pertussis) booster vaccination in healthcare workers in a large academic hospital in Southern Italy in 2022: a cross-sectional study. Front Public Health.

[11] Tuckerman JL, Collins JE, Marshall HS (2015). Factors affecting uptake of recommended immunizations among health care workers in South Australia. Hum Vaccin Immunother.

[12] Eltvedt AK, Poulsen A, Winther TN, Von Linstow ML (2021). Barriers for vaccination of healthcare workers. Hum Vaccin Immunother.

[13] Christodoulakis A, Bouloukaki I, Aravantinou-Karlatou A, Zografakis-Sfakianakis M, Tsiligianni I (2024). Vaccine hesitancy and associated factors amongst health professionals: a scoping review of the published literature. Vaccines (Basel).

[14] Hutin Y, Hauri A, Chiarello L, Catlin M, Stilwell B, Ghebrehiwet T, Garner J (2003). Best infection control practices for intradermal, subcutaneous, and intramuscular needle injections. Bull World Health Organ.

[15] Simard EP, Miller JT, George PA, Wasley A, Alter MJ, Bell BP, Finelli L (2007). Hepatitis B vaccination coverage levels among healthcare workers in the United States, 2002-2003. Infect Control Hosp Epidemiol.

[16] Kumakura S, Shibata H, Onoda K, Nishimura N, Matsuda C, Hirose M (2014). Seroprevalence survey on measles, mumps, rubella and varicella antibodies in healthcare workers in Japan: sex, age, occupational-related differences and vaccine efficacy. Epidemiol Infect.

[17] Celikbas A, Ergonul O, Aksaray S (2006). Measles, rubella, mumps, and varicella seroprevalence among health care workers in Turkey: is prevaccination screening cost-effective?. Am J Infect Control.

[18] Squeri R, Di Pietro A, La Fauci V, Genovese C (2019). Healthcare workers' vaccination at European and Italian level: a narrative review. Acta Biomed.

[19] Fan J, Xu S, Liu Y, Ma X, Cao J, Fan C, Bao S (2023). Influenza vaccination rates among healthcare workers: a systematic review and meta-analysis investigating influencing factors. Front Public Health.

[20] Belay M, Tsega TD, Molla M, Teshome M (2024). Factors associated with COVID-19 vaccine uptake among health professionals in Debre Markos town public health facilities, Northwest Ethiopia. PLOS Glob Public Health.

[21] Sambani C, Muwonge T, Chinyamunyamu L (2025). COVID-19 vaccine uptake, barriers and associated factors among healthcare workers in Malawi. J Public Health Afr.

[22] Getachew T, Lami M, Eyeberu A (2022). Acceptance of COVID-19 vaccine and associated factors among health care workers at public hospitals in Eastern Ethiopia using the health belief model. Front Public Health.

[23] AlShamlan NA, AlOmar RS, AlAbdulKader AM (2024). HPV vaccine uptake, willingness to receive, and causes of vaccine hesitancy: a national study conducted in Saudi Arabia among female healthcare professionals. Int J Womens Health.

[24] Oygar PD, Büyükçam A, Sahbudak Bal Z (2022). Evaluation of vaccination status of health care workers for recommended vaccines and their acceptance of SARS-CoV-2 vaccines. Hum Vaccin Immunother.

[25] Flanagan KL, Fink AL, Plebanski M, Klein SL (2017). Sex and gender differences in the outcomes of vaccination over the life course. Annu Rev Cell Dev Biol.

[26] Algabbani A, AlOmeir O, Algabbani F (2023). Vaccine hesitancy in the Gulf Cooperation Council countries. East Mediterr Health J.

[27] Frati P, La Russa R, Di Fazio N, Del Fante Z, Delogu G, Fineschi V (2021). Compulsory vaccination for healthcare workers in Italy for the prevention of SARS-CoV-2 infection. Vaccines (Basel).

[28] Kyakuwa N, Kimbugwe G, Nakanjako F (2024). High uptake of COVID-19 vaccines among healthcare workers in urban Uganda. PLoS One.

[29] Vo LT, Phan DQ, Tran HG, Nguyen LT, Gyan A, Nguyen HT, Huynh G (2025). Hepatitis B vaccine coverage in health care students: a cross-sectional study in Vietnam. PLoS One.

[30] Davis CL (2019). Targeted Education for the Prevention of Vaccine Refusal (Doctoral Dissertation). https://www.proquest.com/openview/14acf77ea60260fbd1770d88b6022800/1?pq-origsite=gscholar&cbl=18750&diss=y.

[31] Kandiah S, Iheaku O, Farrque M (2022). COVID-19 vaccine hesitancy among health care workers in four health care systems in Atlanta. Open Forum Infect Dis.

[32] Maltezou HC, Dounias G, Rapisarda V, Ledda C (2022). Vaccination policies for healthcare personnel: Current challenges and future perspectives. Vaccine X.

